# Viral tropism is a cornerstone in the spread and spillover of avian influenza viruses

**DOI:** 10.1128/mbio.01690-25

**Published:** 2025-07-14

**Authors:** Nicolas Gaide, Pierre Bessière, Jean-luc Guérin

**Affiliations:** 1 ENVT, INRAE, IHAP, Université de Toulouse 27091, Toulouse, France; Albert Einstein College of Medicine, Bronx, New York, USA

**Keywords:** viral tropism, avian influenza, host adaptation, pathogenesis

## Abstract

In recent years, high pathogenicity avian influenza viruses (HPAIVs) have spread among wild, captive, and domestic birds, as well as mammals. Beyond the resulting economic and ecological losses, spillover into mammals has raised concerns about a potential pandemic. Viral tropism refers to the spectrum of host species, organs, and cells susceptible and permissive to viral infection. It is a potent driver of infection dynamics and shedding patterns, which presents important variations both between and within hosts: in poultry, HPAIV leads to systemic endothelial infection in domestic chickens, whereas neurological and selective epithelial infections are observed in domestic ducks. In mammals, infection can result in respiratory and neurological disease, but the recent outbreaks in domestic dairy cows highlighted a unique and remarkable adaptation to the mammary gland prone to viral shedding in milk. The present review explores viral tropism of HPAIV across recent spillover from birds to mammals and discusses its critical involvement in viral ecology, requiring the constant surveillance and adaptation of control measures.

## INTRODUCTION

Viral tropism can be defined as all living units (i.e., host, organ, tissue, and cellular niches) in which the interaction with a virus leads to functional alteration and eventually perceptible structural alterations. As an information input within a susceptible living unit, viral tropism represents a change in the host activity that supports the completion of viral replication and spread. Viral tropism manifests heterogeneously across living units, creating a dynamic mosaic of susceptible hosts, organs, and cells that drives viral evolution, pathogenesis, and diffusion. Consequently, understanding the viral and host determinants influencing tropism can help in understanding viral ecology, episodic resurgence, and spillover that are raising concerns in terms of pandemic risk.

## A BROAD SPECTRUM OF AVIAN HOSTS

Waterbirds represent the natural reservoir of avian influenza viruses (AIVs), including members of Anseriformes (for instance, *Anatidae* such as ducks, geese, and swan) and Charadriiformes (for instance, *Laridae* and *Scolopacidae* such as gulls, terns, and shorebirds) orders (1). The combination of high intestinal permissiveness and low clinical susceptibility makes wild Anseriformes prone to subclinical infection, AIV shedding, circulation, and environmental diffusion (2–4). Due to migratory patterns, they can spread AIVs over long distances (5). Although AIVs are usually restricted to a few species, A/goose/Guangdong/1/1996-like (Gs/Gd) H5 high pathogenicity avian influenza virus (HPAIV) lineage has been detected in a wide range of wild bird species (6, 7). Following the introduction of AIV into poultry farms, most often by wild birds, both gallinaceous and non-gallinaceous birds can be infected. Domestic Galliformes, including chickens, guinea fowls, turkeys, and quails, are clinically susceptible to HPAIV infection (8–11). In contrast, domestic Anseriformes, such as ducks and geese, display variable clinical outcomes but are considered highly susceptible and shedding species over the course of infection (9, 12). Before 2002, HPAIVs were less pathogenic for domestic Anseriformes, which remained mainly asymptomatic. Since then, Gs/Gd H5 HPAIVs have spread, diversified, and resulted in overall increased pathogenicity and marked neurovirulence in domestic Anseriformes (13, 14). Among ratites, ostriches can be infected with both low pathogenicity AIV (LPAIV) and HPAIV (15, 16). In addition to poultry, domestic Columbiformes, such as racing pigeons, can be infected with H5 HPAIV, leading to mild disease and excretion (17, 18). Indirect transmission can be the result of environmental diffusion and bridging hosts facilitating the introduction of AIV into poultry sites (19–21). Synanthropic wild fauna include a wide range of interacting commensal birds, some of which have been reported to interact with domestic ducks (20, 21). Experimentally, infection of crows and European starlings with LPAIVs or HPAIVs leads to infection, shedding, and seroconversion (22–24). Additionally, birds of prey and scavengers are at risk of exposure through the ingestion of infected prey and carcasses (22, 25, 26).

**Fig 1:**
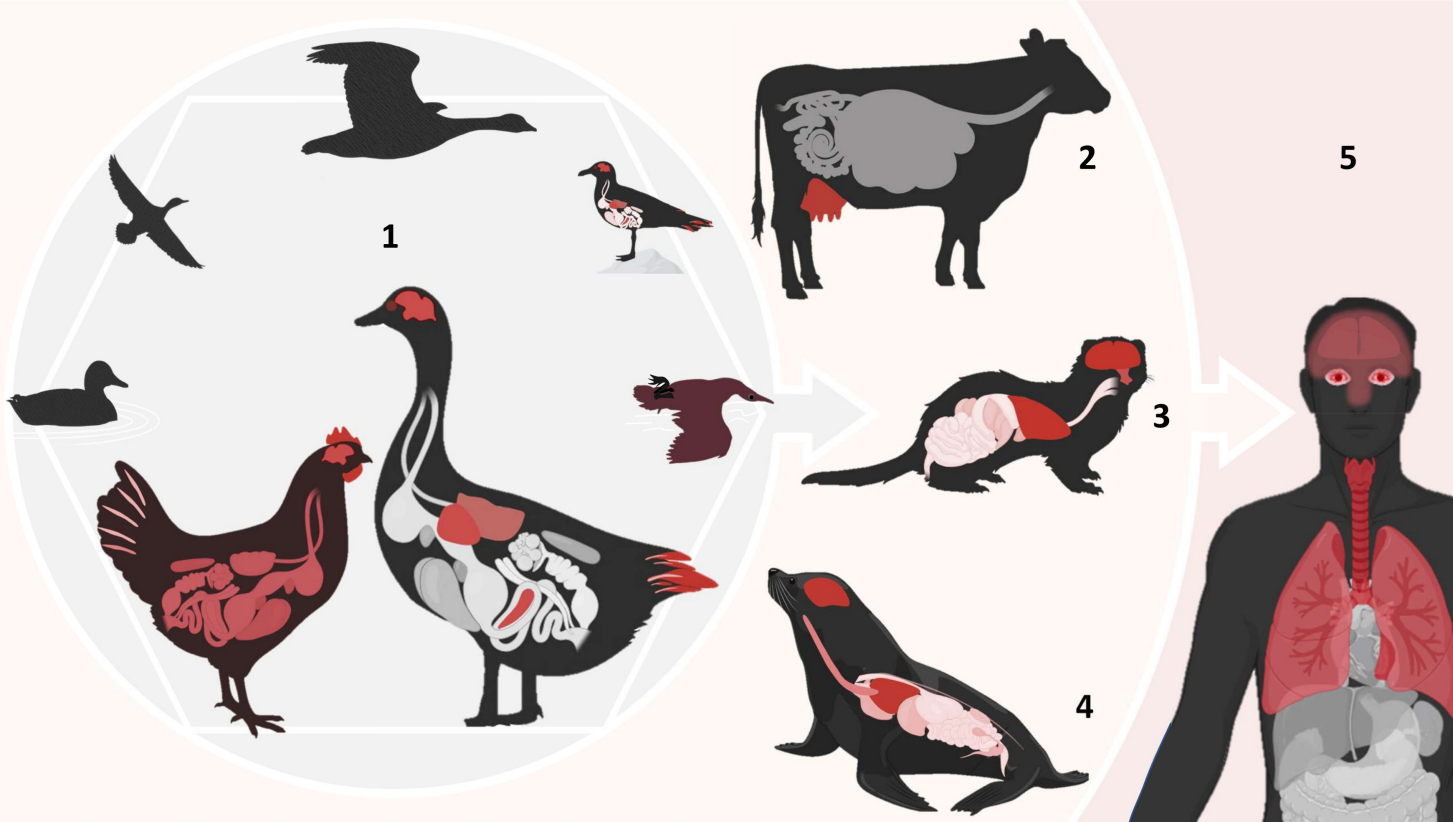
Selective and host-dependent tissue tropism of H5 HPAIVs from birds to mammals. Organs highlighted in red indicate sites of active viral replication and associated lesions. (1) In domestic Anseriformes, such as ducks, Gs/Gd H5 HPAI infection leads to systemic infection that can progress to neurological disease over the course of infection. Tissue pantropism is selective, including marked neurotropism, visceral tropism, and feather epitheliotropism. The infection course in chickens is shorter due to prominent systemic endotheliotropism resulting in systemic fatal acute disease and widespread viral replication. In addition, wild waterbirds carry and spread Gs/Gd H5 HPAIVs, with variable clinical outcomes ranging from asymptomatic to death. (2) In domestic dairy cows, H5 HPAIV presents a remarkable selective tropism for mammary epithelium leading to diffusion into milk. In carnivorous terrestrial (3) and marine (4) mammals, infection is characterized by a marked tropism for respiratory and nervous tissues. (5) In humans, localized forms of Gs/Gd H5 HPAI infection include conjunctivitis as reported in the dairy cow outbreaks in the United States, while severe forms were associated with prominent respiratory tropism and sometimes neurotropism.

Pathology benefits from the accessibility of infected carcasses to investigate viral tropism in both spontaneous and experimental infected birds. Clinical and pathological findings following HPAIV infection result from various factors, including host (e.g., species, breed, and age), viral strain, inoculum (e.g., inoculation titer and route), co-morbidities, and environmental or experimental settings (9, 11–14, 27). In Galliformes, HPAIV infection leads to multi-visceral failure shock as a result of systemic endotheliitis and disseminated intravascular coagulation (11). Grossly, this acute process results in vascular changes (hyperemia and/or cyanosis), subcutaneous edema, and hemorrhages observed in apteric regions of the integument (i.e., wattle, comb, and unfeathered skin), as well as organomegaly, parenchymal necrosis, congestion, edema, and/or hemorrhage (for instance, in the lung, pancreas, and spleen) (11). Necro-inflammatory foci and vasculitis are indeed widespread in the whole body due to the systemic nature of HPAIV endotheliotropism in those susceptible birds (Fig. 1) (9, 11). In contrast with Galliformes, endothelial tropism is limited in ducks (28–30). HPAIV can, however, spread hematogenously, leading to multicentric necrotizing and non-suppurative inflammation. In general, a pathological triad can be observed, including encephalitis, pancreatitis, and myocarditis (Fig. 1). Microscopically, lesions and viral antigens can be detected among viscera and integument (i.e., growing feathers) with prominent but selective tissue tropism for parenchymal and lining epithelium (9, 11, 31, 32).

## A PANZOOTIC AMONG MAMMALS

H5 HPAIV infection has been reported with increased frequency over the past years in a variety of mammal species, ranging from asymptomatic to mild disease or deadly infections (33–35). Marine and terrestrial carnivorous animals were mostly reported, and infection has been associated with scavenging activity and close contact with infected birds (36, 37). H5 HPAIV infection in marine mammals was reported in sea lions, bottlenose dolphins, harbor seals, and grey seals (35, 38–40). Affected terrestrial mammals included several species of bears, foxes, skunk, mink, mountain lion, cats, and bush dogs (33, 34, 41–48). Most of them were not associated with evidence of mammals-to-mammal transmission, although it was suggested for both terrestrial and marine mammals (34, 44, 49). In naturally dead mammals infected with H5 HPAIV, lesions included non-suppurative necrotizing encephalitis, interstitial pneumonia, and vasculitis (Fig. 1) (33, 40, 50, 51). Neurovirulence is a major feature of HPAIV infection in mammals. Viral entry into the central nervous system can occur through hematogenous spread, or ascending neuroinvasion through cranial nerves, such as olfactory nerves, as has been suggested in carnivorous mammals (52–54). A most recent outcome of this adaptation of avian AIVs to mammals is represented by dairy cows, in which viral replication is confined to the mammary epithelium (Fig. 1), causing necrotizing mastitis during the acute stage of infection (55). This unexpected tropism for the mammary epithelium seems to be largely driven by its avian-like sialic acids (SAs) profile and abundance, including α2-3 SAs in alveolar gland and intralobular ducts (56). Other herbivorous animals, such as horses in Mongolia, were later reported to be infected through serological study (57).

## TOWARDS AN INCREASED PANDEMIC RISK

In humans, HPAIV infection was generally associated with close exposure to contaminated animals and carcasses of birds. Eight hundred sixty-eight cases of H5 HPAIV infection were reported over the period 2003–2023 with an overall lethality of 52% (58). Clinical signs included fever, cough, respiratory distress, pneumonia, and, sometimes, neurological signs (59, 60). A pathological investigation conducted on two adults has revealed viral replication within the trachea, alveolar pneumocytes of the lung, brain, small intestine, placenta, and fetus as well as in circulating mononuclear cells, consistent with systemic spread (59). The increased circulation of clade 2.3.4.4b H5N1 viruses in recent years has led to more frequent spillover events, with numerous human cases, particularly in the United States, raising the threat of an emerging virus with pandemic potential (61). Exposure risk is highly dependent on viral tropism that drives viral shedding, as demonstrated by the remarkable epithelial tropism for mammary glands in dairy cows (55). As a consequence, almost all H5N1-infected dairy farm workers, who were exposed to sick cows, developed conjunctivitis (62, 63). In particular, the virus isolated from a farmer’s conjunctiva caused severe infection and lethality in the ferret model, underlining the virus’s ability to adapt to mammals (64). If such infections remained localized, severe cases with the development of severe pneumonia and acute respiratory distress were sporadically and recently reported in Canada and the United States (Fig. 1) (65–67).

## MAJOR DETERMINANTS OF AVIAN INFLUENZA VIRUS TROPISM

Once a host is infected, active viral replication leads to the production of an abundant viral progeny that may exhibit genomic variations: a viral quasi-species (68). This phenomenon can arise from various processes, including replicative mutagenesis promoted by error-prone polymerases, recombination, and genome segment reassortments. Viral quasi-species undergo competition, leading to the selection of the most adapted variants in the given environment (69). This can promote increased viral fitness, immune escape, increased virulence, host adaptation, and jump (69, 70). Consequently, replicative mutagenesis and quasi-species genomic mixing represent a driving force of viral tropism, which is governed by a multifactorial set of determinants acting at different stages of the viral life cycle.

One of the earliest barriers is viral entry, which depends on the interaction between viral hemagglutinin (HA) and host cell surface receptors. HA binds to SAs, which are terminal residues of glycans attached to membrane glycoproteins, glycolipids, or mucus. N-acetylneuraminic acid (Neu5Ac) and N-glycolylneuraminic acid (Neu5Gc) are the most common types, but there are approximately 50 different types of SAs (71). They are attached to underlying sugars (e.g., galactose) of the oligosaccharide chain through a glycosidic bond that can exist in different conformations, such as α2-3 or α2-6 (72).

These conformations are a potent contributor to AIV host range and tropism. Overall, HAs from avian-origin AIVs preferentially bind glycans harboring terminal α2-3 SAs. In contrast, human influenza viruses preferentially bind glycans with terminal α2-6 (73, 74). The distribution and nature of SAs in hosts depend on and vary according to the host species and the organ. For instance, humans predominantly harbor α2-6 SAs in the upper respiratory tract, while α2-3 SAs are mostly expressed in lower portions in alveolar cells and bronchiolar epithelial cells (75, 76). This determines, in part, the lower susceptibility of humans to avian-origin isolates. Among bird species, sialic acid distribution varies notably: geese and ducks predominantly express α2,3-linked receptors throughout the respiratory tract and show minimal α2,6 expression limited to the colon, whereas species like quail exhibit both α2,3 and α2,6 receptors in both the respiratory and intestinal tracts (77).

To be infectious, AIVs require their HA to be enzymatically cleaved. HAs from LPAIVs possess a monobasic cleavage site activated by trypsin-like proteases whose distribution is limited to the respiratory and digestive tracts of birds. This limits infection to localized epithelial tissues. In contrast, HAs from HPAIVs harbor a multibasic cleavage site that allows activation by ubiquitous furin-like proteases, enabling systemic viral spread and tissue pantropism (78–81). Consequently, the viral multibasic cleavage site is a major contributor to HPAIV virulence, by promoting tissue pantropism, systemic disease, and significant mortality (82).

For a virus to cross the species barrier, the adaptation of HA to new SAs is not enough, because the polymerase activity of avian viruses is generally very limited in mammalian cells (83). This is due to an incompatibility between the viral polymerase complex and specific cellular proteins, in particular those of the acidic leucine-rich nuclear phosphoprotein 32 kDa (ANP32) family (84). Mutations in genes encoding the viral polymerase complex can overcome this barrier, such as the PB2 E627K or PB2 D701N mutations (85, 86). These mutations may appear *de novo*, or may already be present in the index host, in the form of a minority variant. For example, clade 2.3.4.4b H5N1 viruses carrying the PB2 D701N mutation are highly virulent in ferrets, even at low doses, and the reversion of this mutation reduces both mortality and airborne transmission (87). Once the HA and the polymerase complex have adapted, a constellation of other mutations favoring replication and transmissibility generally emerges later, as the virus spreads from host to host.

Viral tropism and host adaptation rely on a complex interplay of molecular mechanisms beyond receptor binding and polymerase compatibility. Additional determinants include pH-dependent HA-mediated membrane fusion, efficient import of viral ribonucleoproteins (vRNPs) into the host nucleus, evasion of innate immune responses, a functional balance between HA binding and neuraminidase (NA) cleavage activities, and other factors (88–91). An exhaustive description of these determinants was provided by (92). As the virus replicates in a new host, constellations of mutations can emerge, enabling more efficient replication and host adaptation (93).

## TROPISM AS A MAJOR FACTOR IN SHEDDING AND DIFFUSION PATHWAYS

In permissive hosts, HPAIVs can cause widespread and systemic replication, often leading to high viral loads and substantial environmental shedding. However, this may be counterbalanced by a shortened excretion period due to rapid disease progression and high mortality. For example, chickens are highly susceptible to HPAIV infection, with lower mean death times (MDTs) compared to more resistant species such as ducks (9, 94, 95). In contrast, species that are less clinically susceptible, such as ducks, often exhibit delayed or absent clinical signs, resulting in longer survival times and extended periods of virus replication prior to or without overt disease. Overall, viral tropism, shaped by the interaction between host species and strain virulence, modulates the infection dynamics and influences the balance between pathogenesis and shedding.

Because of their systemic replication, HPAIVs are detected in a wide range of organs. Thus, in the event of an undetected epizootic on a farm, the probability of the virus being present on carcasses after slaughter is greater for HPAIVs than for LPAIVs. Notably, it was through this contamination route that an H5N1 epizootic occurred in Poland, in cats that were fed with contaminated chicken meat (96). In addition, clade 2.3.4.4b H5 HPAIVs exhibit a marked tropism for the feather epithelium in Anseriformes, which can result in the accumulation of infectious viral particles in feather dust (27, 32). Viral replication within growing feather epithelium leads to fragmentation and the release of virus-laden debris (32). While this route of dissemination is less well characterized than fecal or respiratory shedding, it may represent an additional mechanism of environmental contamination and transmission, particularly in settings with high bird density. Its relative contribution to overall transmission dynamics, compared to more established routes, is still unclear and may vary between host species and contexts. However, due to the potential for feather debris to persist in the environment, this pathway could play a role in prolonged environmental contamination and cross-species exposure, including for mammals and humans.

The recent epizootics in dairy cows in the USA are a perfect illustration of the consequences that changes in tropism can have on public health. By replicating in the mammary epithelium, Gs/Gd H5N1 HPAIVs are excreted in large quantities in milk for days: by drinking raw milk, infectious viral particles could come into direct contact with the digestive mucosa, and possibly even the respiratory mucosa, making the consumption of milk an unprecedented source of contamination (55).

## TROPISM AND DIAGNOSTIC METHODS

Similarly, viral tropism conditions the relevance of diagnostic methods. In birds, the standard approach involves collecting oropharyngeal, tracheal, and/or cloacal swabs for real-time reverse transcription-quantitative polymerase chain reaction (RT-qPCR) or viral isolation, in accordance with international guidelines (97). While not yet validated for routine diagnostics, the collection of dust using wipes rubbed against the walls and equipment of poultry buildings, possibly combined with molecules to limit the inhibition of PCR reactions caused by organic material, has been explored as a complementary surveillance, which may allow earlier detection of the virus (19, 98). However, further studies are needed to clarify its sensitivity and utility in different epidemiological contexts. Because viral tropism is different in mammals, the methods used in birds cannot be systematically transposed. For example, during the widespread H5N1 outbreaks on Finnish fur farms in 2023, some infected animals tested negative by RT-qPCR on oropharyngeal swabs, while the virus was detected in brain tissue upon necropsy (99). This likely reflects a progression from initial respiratory infection to systemic and neuroinvasive disease, which is consistent with the neurotropism frequently observed with clade 2.3.4.4b H5Nx viruses in mammals. In such cases, especially during the later stages of infection, necropsy and examination of internal organs, including the brain, may be necessary for definitive diagnosis. However, respiratory or other standard swab specimens remain essential, particularly in early or acute phases of infection. Finally, in H5N1-infected dairy cows, milk is the most relevant sample for virus detection, as there is little to no replication outside the mammary tissue. In addition to individual sampling, novel diagnostic methods highlight the value of wastewater sampling for the detection and monitoring of viral disease (e.g., COVID-19) among the population (100). Wastewater surveillance of H5N1 HPAIV could reveal overall human exposure to contaminated milk and secondary fecal shedding (101).

## CONCLUDING REMARKS

Due to their high genetic diversity and ability to generate new variants, AIVs have a broad host spectrum and episodically across the species barrier. From one species to another, the tissues and cells supporting viral replication can be radically different. Between the feather epitheliotropism in ducks (resulting in the generation of infectious dust), the mammary epitheliotropism in dairy cows (resulting in the excretion of viral particles in milk), and the neurotropism observed in carnivorous mammals (often resulting in an epidemiological dead-end for the virus), control and diagnostic measures, as well as the risk of dissemination, are totally different. Investigating viral tropism in all host species, based on a close collaboration of clinicians, pathologists, and virologists, should therefore be a priority for a better understanding and anticipation of viral emergence.
